# Quantitative simulation of photothermal effect in laser therapy of hypertrophic scar

**DOI:** 10.1111/srt.13305

**Published:** 2023-03-13

**Authors:** Jin Cui Shen, Ya Ya Liang, Wei Li

**Affiliations:** ^1^ Department of Dermatology Chengdu Second People's Hospital Chengdu Sichuan China; ^2^ Tribology Research Institute Key Laboratory for Advanced Technology of Materials of Ministry of Education Southwest Jiaotong University Chengdu China

**Keywords:** histological evolution, hypertrophic scar, laser dose parameter, laser therapy, thermal effect simulation

## Abstract

**Background:**

Laser technology has been widely used in the treatment of hypertrophic scar (HPS). Due to the lack of effective quantitative relationship between laser doses and thermal effect of lesion tissue, the selection of laser doses in clinical laser treatment of HPS is blind, which cannot guarantee the best treatment effect.

**Materials and methods:**

The photothermal model of HPS was established by using finite element method. The effects of laser dose parameters such as laser energy density, pulse width, and spot diameter on the thermal effects of laser treatment were analyzed. According to tissue temperature threshold and thermal damage degree of the simulation results, the optimal laser doses of HPS were selected for the laser treatment experiments of rabbit ear HPSs to verify the rationality of the quantitative photothermal model.

**Results:**

The temperature rise and thermal damage degree of HPS following laser treatment were directly correlated to the laser doses, which grew with the increase of energy density and laser pulse width. For the different spot diameters, the temperature rise decreased with the increase of spot diameter, whereas the thermal damage degree worsened with the increase of spot diameter. Both simulation and experimental results show that the optimal treatment parameters of HPS were as follows: The laser energy density was 7.5 J/cm^3^, the pulse width was 4 ms, and the spot diameter was 7 mm.

**Conclusion:**

The laser dose parameters optimized by the photothermal model have achieved good therapeutic effects in the rabbit ear HPS, indicating that the model can be used for quantitative evaluation of laser doses before clinical treatment.

## 1 INTRODUCTION

Abnormal repair of skin tissue subjected to external trauma will lead to excessive hyperplasia of collagen fibers and vessels to form a dense connective lamellar structure named the early hypertrophic scar (HPS).^1^ At present, laser technology has been widely used in the treatment of HPS and has achieved good therapeutic effects.^2^, ^3^ Its mechanism of action is the principle of selective photothermolysis, whereby light emitted from the pulsed dye laser (PDL) and the CO_2_ laser is absorbed by specific chromophores in the skin (hemoglobin for the PDL laser and water for the CO_2_ laser), which is called laser thermal effect in the tissue.^4^, ^5^, ^6^, ^7^ This thermal effect is mainly impacted by the therapeutic dose and the photothermal characteristics of biological tissues. The research on the photothermal effect of tissue is the theoretical basis for the development of laser therapy technology. However, quantitative research on the laser therapeutic dose is still in its infancy. The clinical treatment is mostly based on the doctor's subjective judgment to determine the therapeutic dose of HPS, which makes laser therapy inevitably ambiguous. In some studies, the finite element method is used to investigate the thermal effect of laser and determine the relationship between the therapeutic dose and the thermal effect of laser, which can improve the predictability and safety of clinical application.^8^, ^9^ The photothermal simulation mainly focuses on heat transfer in tissues, which can be solved by establishing a heat transfer model, to obtain the internal temperature and thermal damage degree of biological tissues. In the early stage, Niemz^8^ previously introduced in detail the estimation of photothermal parameters and thermal damage in laser thermal effect through “Fundamentals and Applications of Laser‐Tissue Interactions.” On this basis, the research on the photothermal effect of skin tissue by means of mathematical simulation calculation has started to be carried out. Lucassen et al.^9^ first comprehensively simulated the laser thermal effect of skin tissue, proposed the distribution of laser photons in skin tissue by using the Monte Carlo method, and further calculated the temperature change inside the tissue. With the continuous improvement of the existing theories, Shafirstein et al.^10^ used the two‐dimensional diffusion approximation theory to analyze the distribution of laser energy in the tissue and showed the temperature distribution in the skin tissue through the two‐dimensional heat conduction equation, taking more into account the influence of blood perfusion rate in tissues on laser thermal effect.

Although the simulation results on the laser thermal effect are abundant, there are few studies on estimating the therapeutic dose of scar using the photothermal model. Studies have demonstrated that skin tissue is an optically turbid medium, and lesions will change its physiological characteristics, resulting in various changes in its optical and physical properties.^11^ Through the simulation of laser thermal effect in pathological skin, the conduction of laser in the pathological tissue can be better understood. By simulating different laser treatment doses, the safety of clinical treatment can be adjusted and predicted according to the tissue temperature threshold and the degree of thermal damage. In this paper, a photothermal model of HPS was established by using finite element method based on the existing research. The effects of laser dose parameters such as laser energy density, pulse width, and spot diameter on the thermal effect of laser treatment were analyzed. Then, the laser treatment doses of HPS optimized from the simulation results were used as the treatment parameters, and the laser treatment experiments were carried out with the HPS model of rabbit ear to verify the rationality of the quantitative photothermal model. The optimal treatment scheme of HPS through the quantitative analysis of photothermal model is expected to provide guidance for the selection of dose parameters in clinical laser therapy.

## 2 MATERIALS AND METHODS

### 2.1 Photothermal simulation method

COMSOL Multiphysics simulation software was used to establish the simple 3D models of HPS and normal skin under the condition of keeping the optical parameters unchanged during laser radiation. The laser heat source term was determined according to the Gaussian distribution characteristic of laser beam. Biological heat transfer equation was used to establish the law of energy conservation in tissue. The optimal laser treatment doses were selected according to the thermal damage theory. The theories and method of photothermal simulation are as follows.

#### 2.1.1 Laser heat source

As a turbid medium, skin tissue has a scattering reaction when absorbing laser energy, which would decay exponentially with the incident depth. The photothermal effect of the biological tissue is that tissue absorbs laser energy and converts it into heat energy. Laser heat source (*Q*) is the sedimentation of laser energy in the tissue, which follows the law of radial Gaussian distribution and axial exponential attenuation. Its calculation method is shown in the following equations^12^:

(1) $$Q=\mu_{a}\left(1-R\right)\phi_{0}\exp\left[-0.5\frac{r^{2}}{\omega_{0}}\exp\left(-\mu_{s}z\right)-\mu_{t}z\right]\exp\left[-4\frac{{\left(t-\tau \right)}^{2}}{\tau^{2}}\right]$$

(2) $$\phi_{0}=\frac{2P}{\pi \omega_{0}^{2}}$$

(3) $$\mu_{t}=\mu_{s}+\mu_{a}$$

where *μ_a_* is the absorption coefficient, *μ_s_* is the scattering coefficient, *μ_t_* is the sum of the absorption and scattering coefficients of the tissue, *R* is the reflectivit*y, Φ*_0_ *is th*e luminous flux density, *r* and *z* are the fitting parameters, *τ* is the laser pulse width (irradiation time), *P* is the laser power, and *ω*_0_ is the laser spot diameter. Based on Tseng's research results on photothermal parameters of normal skin and scar, the relevant parameters of the tissues were obtained by using GetData software, as shown in Table 1.^12^

**TABLE 1:** The optical parameters of tissue.^12^

	Normal skin	Scar
*u_s_* (cm^−1^)	183.92	118.37
*u_a_* (cm^−1^)	1.395	5.574
*R* (cm^−1^)	0.58	0.2
*ρ_b_* (kg/m^3^)	1000	1000
*c_b_* (J/(kg K))	4180	4180
*w_b_* (1/s)	6.4e − 3	6.4e − 3
*T*_0_ (°C)	37	37
*T_art_* (°C)	37	37
*T*_1_ (°C)	25	25

#### 2.1.2 Biological heat transfer equation

The heat transfer of skin is simulated by using the Pennes equation, which is the most basic equation for heat transfer of biological tissue.^13^ The solution of biological heat transfer equation is to analyze the conduction process of laser heat in biological tissue, and its main form is shown in the following equation^12^:

(4) $$\rho c\frac{\partial T}{\partial \tau }=\nabla \cdot \left(k\nabla T\right)+\rho_{b}c_{b}w_{b}\left[T_{art}-T\right]+Q$$

where *ρ* is the density of tissue, *c* is the special heat capacity of tissue, *T* is the temperature of tissue, *k* is the thermal conductivity of tissue, *p_b_* is the density of blood, *c_b_* is the special heat capacity of blood, *w_b_* is the blood perfusion rate, *T_art_* is the arterial temperature, *Q* is the laser heat source, and *τ* is the laser pulse width.

#### 2.1.3 Thermal damage theory

The thermal damage of biological tissues is a chemical process. When the temperature exceeds the tolerance threshold of the tissue, irreversible damage occurs inside the tissue that is the thermal damage. In 1972, Moritz and Henriques have put forward the Arrhenius dynamic theory, which described the damage degree by the proportion of tissue heat damaged volume.^14^ The calculation form is shown in the following equation^14^:

(5) $$\Omega =\int_{0}^{t}A\exp\left(-\frac{E_{a}}{RT}\right)dt$$

where *A* is the frequency factor (*A* = 4.575 × 10^72^), *E_a_* is the activation energy of tissue (*E_a_* = 4.71 × 10^5^ J/mol), *R* is the general gas constant (*R* = 8.314 J/(mol K)), *T* is the temperature of tissue, and *t* is the heat time. At present, the common clinical burn measurement values are as follows: first degree burn *Ω* = 0.58, second degree burn *Ω* = 1.0, and third degree burn *Ω* = 10^4^.

#### 2.1.4 Finite element model of HPS

**FIGURE 1:**
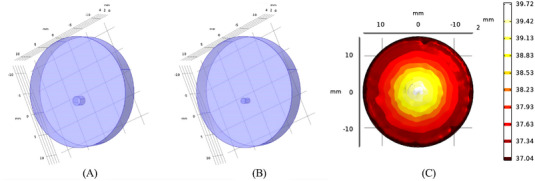
Tissue finite element model: (A) hypertrophic scar (HPS), (B) normal skin, and (C) typical temperature distribution in skin tissue.

The HPS and normal skin were simplified as a medium with constant optical parameters in the process of laser radiation. In the simulation software of COMSOL Multiphysics, the skin was defined as a cylinder model with a diameter of 20 mm and a thickness of 4 mm, ignoring the influence of boundary conditions. Studies have shown that the average density of blood vessels in normal skin is about 1.72 ± 0.608/cm^2^, whereas that in HPS is about 2.92 ± 1.293/cm^2^.^15^ Compared with the normal skin, the blood vessels in HPS are thicker, strip shaped, and vertical to the epidermal surface, with an average diameter of about 73.14 ± 13.8 um.^15^ In this simulation study, the blood vessels in normal skin and HPS were simplified into cylinders with diameters of 0.6 and 1.0 mm, and height of 2.0 mm respectively, as shown in Figure 1A,B. The boundary of the HPS which in contact with ambient was defined as the thermal convection, and other boundaries were set as the constant temperature state (*T* = 37°C). The thermal effect simulation parameters were as follows: laser energy density *q* (4, 7.5, and 10 J/cm^3^), laser pulse width *τ* (0.4, 4, and 10 ms), and laser spot diameter *ω*_0_ (5, 6, and 7 mm). The optimal laser doses were determined according to the simulation results of the above parameters. The conversion formula of different laser treatment doses and laser heat source term is as follows:

(6) W=q·s

(7) $$P=\frac{W}{\tau }$$

where *W* is the single pulse energy, *s* is the laser spot area, and *P* is the power.

### 2.2 Establishment of HPS model

**FIGURE 2:**
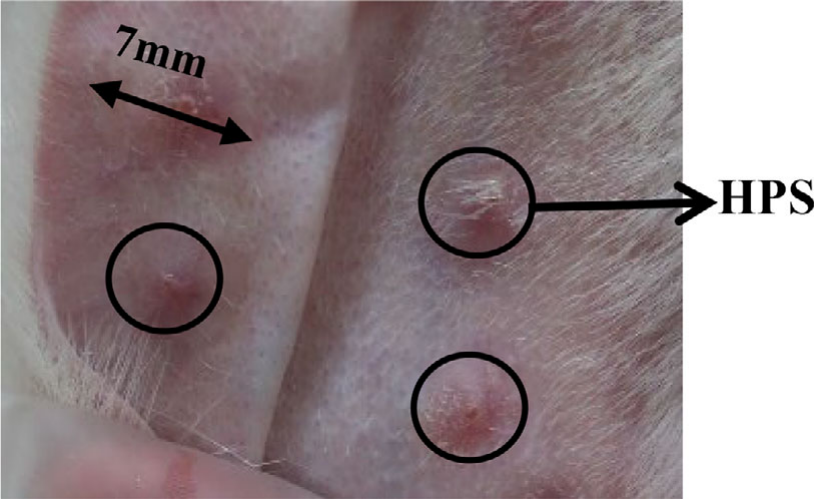
Hypertrophic scar (HPS) models.

Rabbit ear skin was selected to simulate human skin. All rabbit experiments have passed the medical ethics review and were conducted following the ethical standards stipulated in the Declaration of Helsinki. The rabbits were supplied by Experimental Animal Culture Center, Sichuan province. All experimental procedures were performed in compliance with regulations for the Administration of Affairs concerning Experimental Animals, China. Fifteen healthy New Zealand rabbits with a weight of 2–2.5 kg were selected for the study with the approval of the Institutional Animal Care and Use Committee, China. First, 13 healthy New Zealand rabbits were anesthetized by intra‐abdominal injection of 3% sodium pentobarbital (1 mL/kg). Figure 2 shows four round wounds with a diameter of 10 mm made using a CO_2_ laser to ablate ear tissue. Postoperatively, the wounds were washed with iodophor once daily for a week. The rabbits were kept in cages for 35 days to allow for wound healing and HPS formation. As the size of the wound would shrink after healing, a total of 90 HPS models with diameters of 6–8 mm were selected for laser treatment (Figure 2). The remaining two healthy New Zealand rabbits were analyzed as normal skin samples.

### 2.3 Laser treatment experiment

**FIGURE 3:**
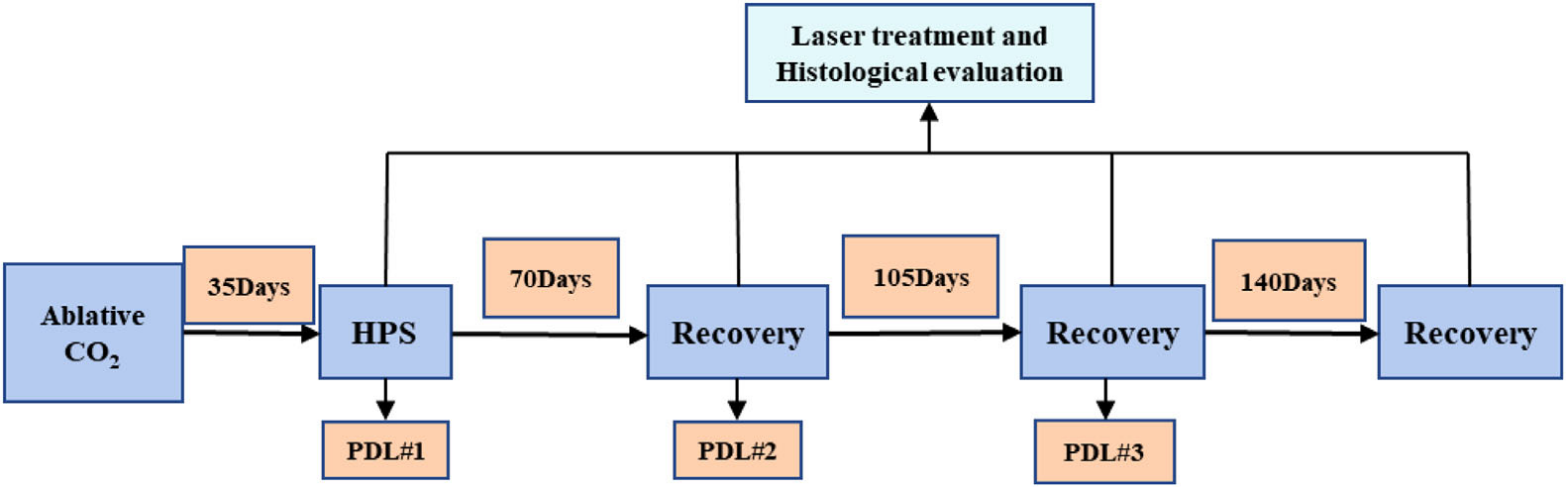
Experimental process and timeline.

**FIGURE 4:**
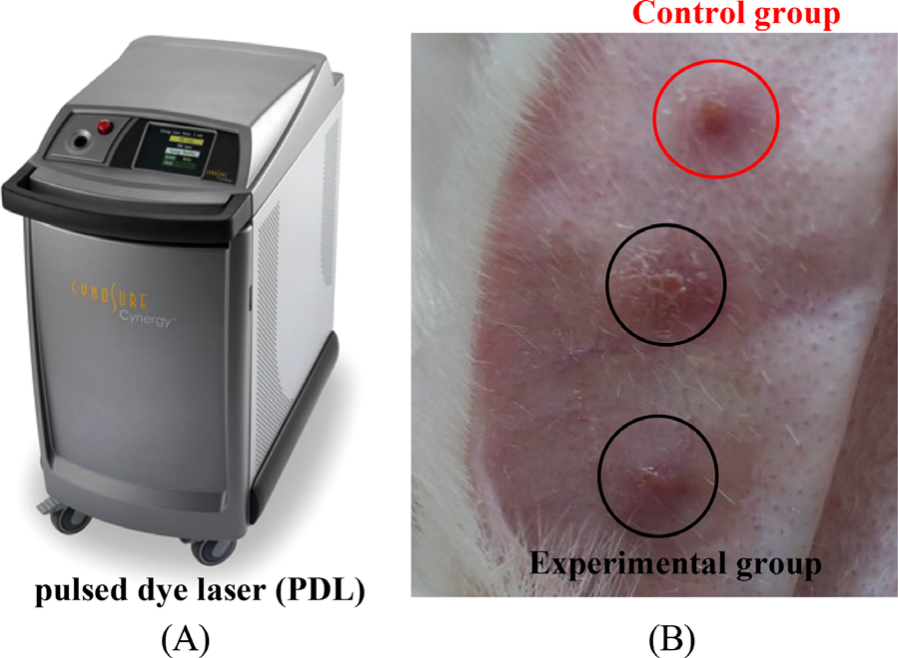
Laser treatment: (A) laser equipment and (B) the morphologies of hypertrophic scar (HPS).

The experimental process and timeline of laser treatment are shown in Figure 3. Eighty HPSs were treated with PDL for three consecutive times with an interval of 35 days between laser treatments. Ten HPSs were used as blank control group. The PDL equipment (Cynergy, USA) used in this laser treatment experiment is shown in Figure 4A. The built‐in parameters of the PDL equipment are as follows: Standard wavelength is 585 nm, pulse width is 0.5–40 ms, spot diameter is 5, 7, 10, and 12 mm, and maximum repetition frequency is 2 Hz. The PDL laser treatments were conducted in the following manner: After the rabbit was anesthetized, a healthy New Zealand rabbit ear was fixed on the PDL laser test‐bed with bandages. The treatment parameters were as follows: Pulse width was 4 ms, PDL energy density was 4, 7.5, and 10 J/cm^3^, and spot diameter was 7 mm. Each HPS was irradiated once by laser spot under the same laser treatment parameters, and the morphologies of HPSs in the laser treated experimental group and the control group are shown in Figure 4B. Table 2 lists the sample numbers for the three consecutive laser treatments with an interval of 35 days and the sample numbers for histological analyses. After each laser treatment, 10 HPSs were selected for the histological analysis. For comparative analysis, the histological analysis was also performed on 10 HPSs from the control group and 10 normal skin samples.

**TABLE 2:** Experimental sample data

Sample quantity	Normal skin	HPS	PDL 1	PDL 2	PDL 3
Therapy	0	0	80	70	60
Histology	10	10	10	10	10
Control group	10	10	10	10	10

Abbreviations: HPS, hypertrophic scar; PDL, pulsed dye laser.

### 2.4 Histological evaluation

Through histological analysis, the histological changes of HPS after laser treatment can be studied to verify the simulation effect of laser quantification parameters. After the rabbits were anesthetized and disinfected again, tissues with a diameter of 5 mm were removed with a trephine. The histological sections were prepared according to the routine method of dermatopathology.^16^, ^17^ Hematoxylin and eosin staining, van Gieson staining, and Masson staining were used to observe the microstructure of HPS, and the structure and quantity of collagen and elastic fibers in tissue were quantitatively analyzed. Tissue sections were observed with a biomicroscope (BX63, Olympus, Japan). The tissue occupied by collagen fibers and the total area of HPS tissue were measured by using ImageJ software of BX63. The proportion of collagen fiber was calculated by the following equation:

(8) $$\begin{array}{ccc} & & \text{Proportion of collagen fibers}=(\text{collagen fiber area})/\\ & & \;(\text{total tissue area})\times 100\% \end{array}$$

### 2.5 Statistical analysis

All test data are presented as mean and standard deviation. *F*‐test was used to determine the significant differences among samples under the same conditions. The independent *T* test was used to compare the thickness changes of HPS under different laser parameters, and the statistical significance level was *p* < 0.05.

## 3 RESULTS

### 3.1 Simulation results of photothermal effect

**FIGURE 5:**
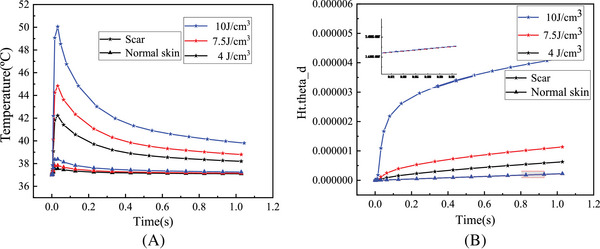
The changes of laser thermal effect with time in normal skin and hypertrophic scar (HPS) at different energy densities *q*: (A) central temperature and (B) proportion of thermal damage volume.

**FIGURE 6:**
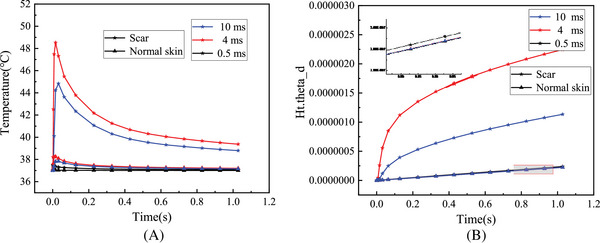
The changes of laser thermal effect with time in normal skin and hypertrophic scar (HPS) at different pulse widths *τ*: (A) central temperature and (B) proportion of thermal damage volume.

**FIGURE 7:**
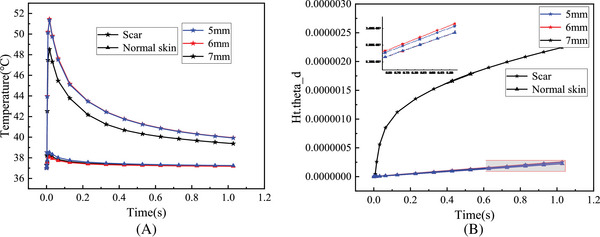
The changes of laser thermal effect with time in normal skin and hypertrophic scar (HPS) at different spot diameters *ω*0: (A) central temperature and (B) proportion of thermal damage volume.

Figure 1C shows the typical simulation result of temperature distribution in skin tissue when pulse width *τ* was 4 ms, spot diameter *ω*_0_ was 7 mm, and energy density *q* was 7.5 J/cm^3^. It is obvious that the central temperature of the skin tissue was the highest, and the temperature dropped slightly from the center to the outside. The changes of the central temperature of normal skin and HPS with time at different energy densities *q* are shown in Figure 5A. The central temperature of all skin tissues elevated with the increase of energy density *q* and decreased with the increase of laser irradiation time. Compared with the normal skin, the central temperature of HPS changed greatly with the increase of the energy density *q* and can reach 52°C at the energy density *q* of 10 J/cm^3^, whereas the central temperature of the normal skin was only 38.5°C. After 1 s, the central temperature of HPS dropped to 40°C, which was still higher than the normal body temperature. As can be seen from Figure 5B, the proportion of skin tissue thermal damage volume gradually increased with duration at different energy densities *q*. It is obvious that the thermal damage degree of HPS became serious with the increase of energy density *q*, especially at the dose of 10 J/cm^3^, the tissue thermal damage degree increased significantly. In contrast, the normal skin had less thermal damage and was not sensitive to the change of energy density *q* (*p* > 0.05). Figure 6 shows the changes of central temperature and the proportion of thermal damage volume of normal skin and HPS with different pulse widths *τ* as a function of laser irradiation time. The central temperature of skin tissue decreased with time, and the degree of thermal damage became more serious with time. Compared with the normal skin, the central temperature and the degree of thermal damage of HPS changed significantly with the increase of pulse width *τ*. When the pulse width *τ* was 4 ms, the central temperature and the degree of thermal damage of HPS were the highest. However, the central temperature and the degree of thermal damage of the normal skin did not change significantly with the increase of pulse width *τ* (*p* > 0.05), which was the same as that of different energy densities *q*. Figure 7 shows the changes of central temperature and proportion of thermal damage volume of normal skin and HPS with laser irradiation time at different laser spot diameters *ω*_0_. With the increase of spot diameter *ω*_0_, the central temperature of HPS decreased gradually, whereas the degree of thermal damage increased. Moreover, the thermal damage of HPS was much greater than that of other parameters at the spot diameter of 7 mm. Different from HPS, the thermal effect of normal skin was not significantly correlated with the spot diameter (*p* > 0.05).

### 3.2 Morphological changes of HPS after laser treatment

**FIGURE 8:**
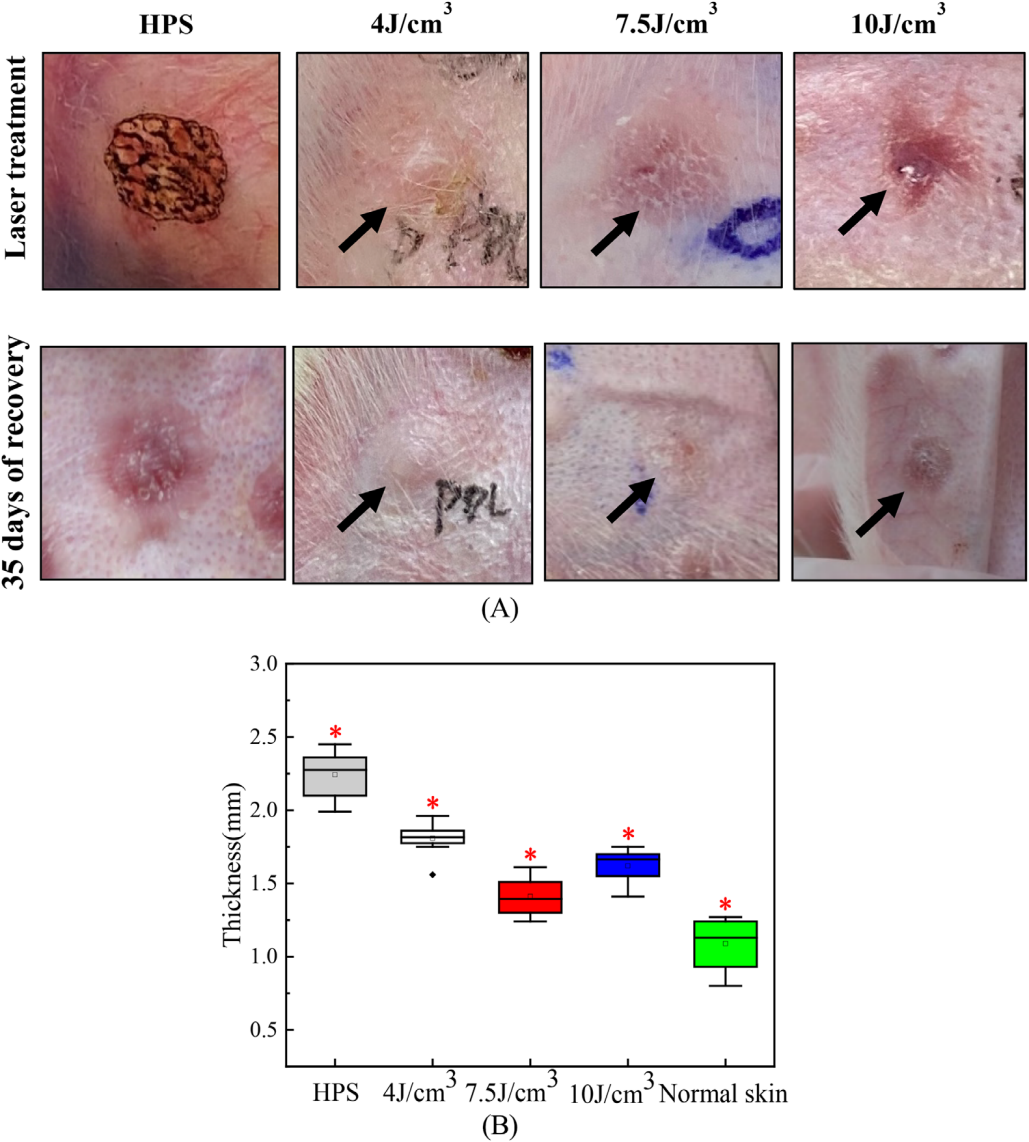
Morphological changes after laser treatment: (A) the evolution of hypertrophic scar (HPS) surface morphology and (B) the change of average thickness.

The HPSs of rabbit ears were treated with the energy density of 4–10 J/cm^3^, the pulse width of 4 ms, and the spot diameter of 7 mm. Figure 8A shows the morphology evolution of the HPSs after 35 days of recovery, and Figure 8B shows the average thickness of rabbit ears corresponding to the HPSs treated with different energy densities. It is evident that the surface of untreated HPS was flushed and uneven, forming folds. The average thickness of untreated HPS was about 2.24 ± 0.15 mm, which was twice that of normal skin. After 35 days of laser treatment with different energy densities, the redness, swelling, and folds on the surface of HPS tissues disappeared. Especially at the energy density of 7.5 J/cm^3^, the characteristics of HPS were significantly weakened after three consecutive laser treatments, and the tissue became soft and flat, close to the normal skin surface. At this time, the average thickness was 1.43 ± 0.12 mm, which was close to the thickness of normal skin. When the energy density was 4 J/cm^3^, obvious HPS characteristics could still be observed on the surface of rabbit ear. When the energy density was 10 J/cm^3^, it was found that the surface edema of HPS was obvious, and pigmentation appeared on the surface of rabbit ear after the recovery. The average thickness of the latter two energy densities was higher than that of the energy density of 7.5 J/cm^3^ (*p* > 0.05).

### 3.3 Histological changes after laser treatment

**FIGURE 9:**
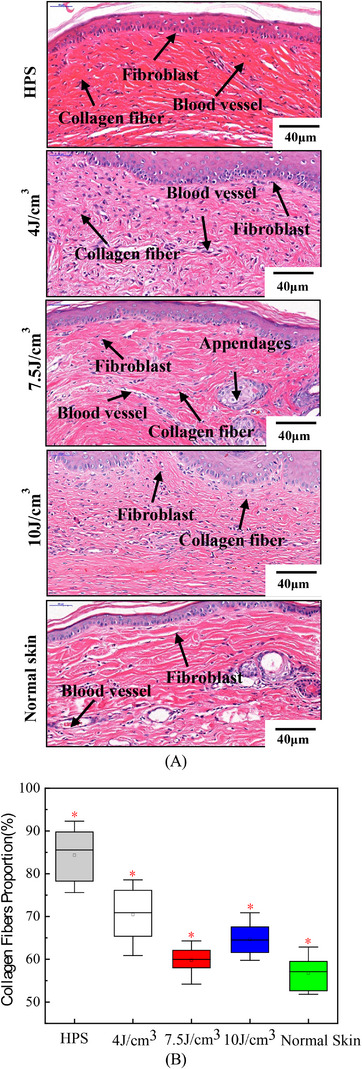
Histological changes after laser treatment: (A) typical histological micrographs of normal skin 40 µm fibroblast, blood vessel; 40 µm hypertrophic scar (HPS), blood vessel, fibroblast collagen fiber; 10 J/cm^3^ 40 µm collagen fiber, fibroblast; 4 J/cm^3^ 40 µm collagen fiber, blood vessel, fibroblast; 7.5 J/cm^3^ 40 µm fibroblast, appendages, blood vessel, collagen fiber tissue by VG and Masson staining and (B) the change of the average proportion of collagen fiber.

Figure 9A reveals the typical histological micrographs of HPSs and normal skin following the different laser energy densities, and Figure 9B shows the corresponding changes of the average proportion of collagen fiber in tissues after laser treatment. The normal skin contains hair follicles, nerves, and other appendages. The collagen fibers were organized parallel to the tissue surface in a wavy yet orderly arrangement. Following HPS formation, the collagen fibers were more abundant, arranged irregularly, and present in the form of short and curly shapes. After treatment with different laser energy densities, the collagen fibers in HPSs were reduced significantly in number, whereas the number of elastic fibers increased gradually. As can be seen from Figure 9B, the content of collagen fibers in untreated HPS was as high as 84%, whereas that in normal skin tissue was about 56%. Comparing the content of collagen fibers in treated HPSs following different laser doses, it was found that when the energy density was 7.5 J/cm^3^, the arrangement and content of collagen fibers of HPSs were close to that of normal skin (*p* < 0.05).

## 4 DISCUSSION

In the present study, the laser treatment parameters of HPS were quantitatively simulated by using the photothermal model of HPS, and the thermal effects of different laser energy density *q*, pulse width *τ*, and spot diameter *ω*_0_ in laser treatment were analyzed. The optimal laser doses were determined according to the tissue temperature threshold and the proportion of thermal damage volume. Finally, the effectiveness of the photothermal model was further verified by the laser treatment experiments of rabbit ear HPSs.

The simulation results of thermal effect in Figures 5, 6, 7 show that there were significant differences in the internal temperature rise and thermal damage degree between HPS and normal skin tissue under the same laser dose parameters. As the HPS does not have the same heat dissipation function as normal skin, its absorption degree of laser energy was significantly greater than that of normal skin. As a result, in the process of laser irradiation, the HPS can absorb more laser energy, thereby generating more heat inside the tissue. In this way, the internal temperature rise and thermal damage degree of HPS were greater than those of normal skin under the same laser doses. These simulation results were consistent with the selective photothermal principle proposed by Rox Anderson et al.,^4^ that is, the absorption of laser energy by the scar skin is much greater than that of the normal skin, thus achieving good laser treatment effects.

Furthermore, the simulation results show that the temperature variation and thermal damage of HPS following laser treatment were directly correlated to the laser doses. However, the variation of thermal effect caused by each parameter was slightly different. The central temperature and the thermal damage degree of HPS all grew with the increase of energy density *q* (Figure 5). For the different laser pulse widths *τ*, the thermal effect of HPS changed slightly differently, which first increased and then decreased with the increase of laser pulse width, and was the highest at the pulse width of 4 ms (Figure 6). When the laser pulse width was 0.5 ms, the irradiation time was less than the thermal relaxation time of HPS, that is, the laser energy will suddenly disappear before the thermochemical reaction in the tissue began during laser irradiation, so there was basically no temperature rise in the tissue. When the laser pulse width increased to 4 ms, the heat would be transferred from the central tissue of HPS to the surrounding tissue, causing damage to the surrounding tissue, and the overall thermal damage degree of HPS was relatively high. When the laser pulse width was 10 ms, the tissue thermal response and its own heat dissipation system would start at the same time after a long time of irradiation, so that the internal temperature rise and thermal damage degree were less than that of the laser pulse width of 4 ms. For the different spot diameters *ω*_0_, the smaller the laser spot diameter, the more concentrated the energy, and the more obvious the central temperature rise of the tissue (Figure 7A). However, when the spot diameter increased, the heating area of HPS also expanded accordingly, leading to the increase of the proportion of thermal damaged tissue. Therefore, the thermal damage degree of HPS worsened with the increase of spot diameter (Figure 7B).

In general, the laser treatment of HPS cannot be focused on higher doses, and the reasonable parameters can not only produce the good treatment effect but also have little impact on the surrounding normal tissues. The previous simulation results demonstrate that the internal damage in HPS was less than third degree burn. In order to ensure a better laser treatment effect, the maximum proportion of thermal damage volume should be selected while considering the temperature threshold. Studies have shown that when the temperature of biological tissue rises to about 50°C, the protein in the tissue will undergo denaturation, resulting in tissue coagulation necrosis.^4^ Therefore, the temperature threshold of HPS was set to 50°C. Under this limitation, the optimal treatment parameters obtained by comparing the simulation results are as follows: The laser energy density *q* was 7.5 J/cm^3^, the pulse width was *τ* 4 ms, and the spot diameter *ω*_0_ was 7 mm.

In addition, Figures 8 and 9 reveal that after three consecutive PDL laser treatments with energy density of 4–10 J/cm^3^, pulse width of 4 ms, and spot diameter of 7 mm, the surface characteristics of HPS such as flushing and folding gradually disappeared, the direction of fibers in the tissue gradually parallel to the epidermis, and the number of collagen fibers decreased significantly. Especially at the energy density of 7.5 J/cm^3^, the surface of HPS became flat, and the color, texture, and thickness of HPS gradually approach that of normal skin tissue. The proportion of collagen fibers in HPS tissue gradually decreased to 60% with a more normal arrangement, which was gradually close to normal skin. The experimental results further validated that the therapeutic doses optimized by our photothermal model had a significant effect on HPS, which indicated the rationality and feasibility of the analysis of the photothermal model. The present work provides basic research for establishing the quantitative relationship between the structure parameters of HPS tissue and hyperthermia parameters in clinical practice.

## 5 CONCLUSION

1. The simulation results of photothermal model revealed that the thermal effect of HPS was significantly correlated with laser doses. The temperature rise and thermal damage degree of HPS increased with the increase of energy density and laser pulse width. For the different spot diameters, the temperature rise decreased with the increase of spot diameter, whereas the thermal damage degree worsened with the increase of spot diameter.
2. The histomorphology results showed that the laser dose parameters optimized by the photothermal model have achieved good therapeutic effects in the rabbit ear HPS.
3. The photothermal model can quantitatively analyze the effect of laser doses on the thermal effect of HPS and can be used for the selection of laser dose parameters and safety evaluation before clinical treatment.

## CONFLICT OF INTEREST STATEMENT

All authors have no conflict of interests to disclose.

## Data Availability

The data that support the findings of this study are available from the corresponding author upon reasonable request.
